# Visualizing the Entire Range of Noncovalent Interactions in Nanocrystalline Hybrid Materials Using 3D Electron Diffraction

**DOI:** 10.1021/jacs.2c02426

**Published:** 2022-06-09

**Authors:** Yi Luo, Max T. B. Clabbers, Jian Qiao, Zhiqing Yuan, Weimin Yang, Xiaodong Zou

**Affiliations:** † Department of Materials and Environmental Chemistry, Stockholm University, SE-106 91 Stockholm, Sweden; ‡ State Key Laboratory of Green Chemical Engineering and Industrial Catalysis, Sinopec Shanghai Research Institute of Petrochemical Technology, 1658 Pudong Beilu, Shanghai 201208, China

## Abstract

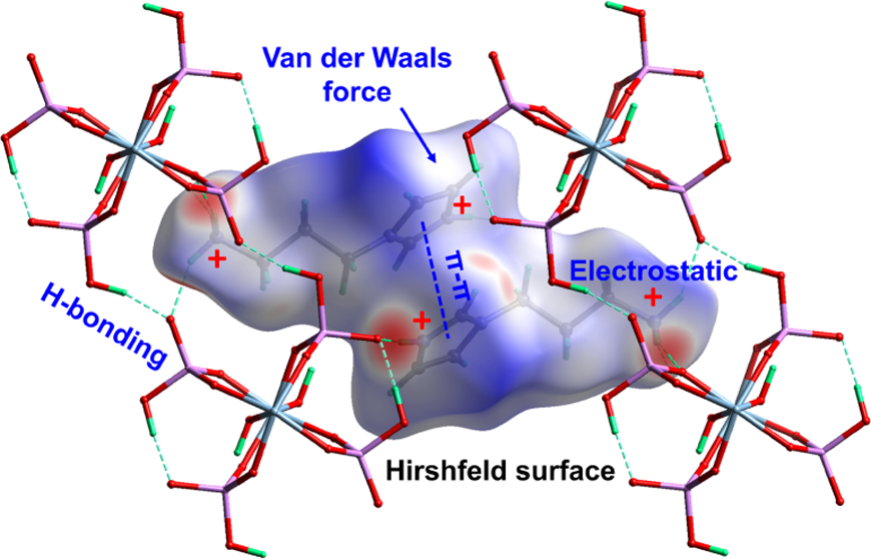

Noncovalent interactions are essential in the formation and properties of a diverse range of hybrid materials. However, reliably identifying the noncovalent interactions in nanocrystalline materials remains challenging using conventional methods such as X-ray diffraction and spectroscopy. Here, we demonstrate that accurate atomic positions including hydrogen atoms can be determined using three-dimensional electron diffraction (3D ED), from which the entire range of noncovalent interactions in a nanocrystalline aluminophosphate hybrid material SCM-34 are directly visualized. The protonation states of both the inorganic and organic components in SCM-34 are determined from the hydrogen positions. All noncovalent interactions, including hydrogen-bonding, electrostatic, π–π stacking, and van der Waals interactions, are unambiguously identified, which provides detailed insights into the formation of the material. The 3D ED data also allow us to distinguish different types of covalent bonds based on their bond lengths and to identify an elongated terminal P=O π-bond caused by noncovalent interactions. Our results show that 3D ED can be a powerful tool for resolving detailed noncovalent interactions in nanocrystalline materials. This can improve our understanding of hybrid systems and guide the development of novel functional materials.

## Introduction

Noncovalent interactions, including electrostatic, hydrogen-bonding, π–π stacking, and van der Waals interactions, are at the core of supramolecular chemistry.^1−4^ Although these noncovalent interactions are relatively weak compared to covalent bonding, they play a vital role in the formation and chemical processes of functional materials.^5^ Noncovalent interactions have been utilized in the development of important functional materials such as catalysts^6,7^ and adsorbents.^8−10^ They are also used for tailoring crystallization,^11^ designing molecular machines,^12^ and studying host–guest interactions for biomedical applications.^13,14^ Novel strategies based on noncovalent interactions have been developed for the synthesis of advanced functional materials.^15−17^ For example, porous materials such as zeolites, metal-organic frameworks (MOFs), covalent organic frameworks (COFs), and hydrogen-bonded organic frameworks (HOFs) are synthesized through noncovalent interactions.^18−22^ Zeolites are commonly synthesized using organic structure-directing agents, which can introduce electrostatic, hydrogen-bonding, and/or van der Waals interactions to direct the formation of zeolite frameworks.^17,18^ MOFs are typically organic–inorganic hybrids built on the coordination interactions between the metal nodes and organic linkers.^19^ COFs are constructed via covalent bonds, and noncovalent interactions (such as π–π stacking) are also present in some COFs.^21^ HOFs are assembled from organic building blocks by means of hydrogen-bonding interactions.^22^ The flexibility and wide variety of noncovalent interactions make these materials highly tunable, enabling the development of numerous novel framework materials.^17−22^ Reliably identifying the entire range of noncovalent interactions involved in crystalline material formation is therefore crucial in the development of novel functional materials and supramolecular chemistry.

Spectroscopy techniques such as nuclear magnetic resonance (NMR), infrared, ultraviolet–visible, and Raman spectroscopy are used to characterize noncovalent interactions.^23−25^ However, these techniques are limited by the specified signal channel, spatial resolution, and signal-to-noise ratio of the spectra. The results can therefore be ambiguous, and typically only partial noncovalent interactions can be resolved even when different spectroscopy techniques are combined.

Single-crystal X-ray diffraction (SCXRD) is mostly used to determine three-dimensional (3D) atomic structures and resolve noncovalent interactions in crystalline materials,^7,26,27^ which enables the understanding of how noncovalent interactions affect covalent bond lengths and angles.^28^ However, SCXRD requires relatively large (>5 × 5 × 5 μm^3^) and well-ordered crystalline materials. Growing large crystals suitable for SCXRD can be challenging and time-consuming, especially for crystals that are formed via relatively weak noncovalent interactions. These factors can complicate, or even prohibit structure determination of such materials by SCXRD.

Alternatively, powder X-ray diffraction (PXRD) can be used to gain structural insights from samples composed of small micron- or nanometer-sized crystals. However, structure determination by PXRD often requires phase-pure samples, and peak overlapping in the one-dimensional pattern can make structure determination difficult.^29^ The noncovalent interactions resolved from PXRD data are therefore often ambiguous, owing to the large number of restraints that are required in structure refinement.

Compared to X-rays, neutrons are scattered by the nuclei of the atoms and have a unique advantage in locating hydrogen positions.^30−34^ Single-crystal neutron diffraction (SCND) has been used for studying hydrogen-bonding interactions in a wide range of materials.^30−33^ A drawback is that the crystal size required for SCND is even larger than that needed for SCXRD.^32^ Hydrogen positions can also be determined by neutron powder diffraction; however, it is limited to crystals with relatively small unit cells where peak overlapping is not severe.^33^ In addition, sample deuteration is often required for neutron powder diffraction, which can be both time-consuming and chemically challenging.^33^

Electrons are scattered by both the electrons and nuclei of the atoms and have 10^4^ times stronger interactions and significantly lower radiation damage per event compared to X-rays.^34^ This enables the use of electron diffraction for the structure determination of nanocrystalline inorganic and organic materials too small for X-ray and neutron diffraction.^35^ Electrons are more sensitive toward hydrogen atoms relative to X-rays, facilitating the location of individual hydrogen atoms in organic and inorganic samples at a subatomic resolution.^36−41^ Three-dimensional electron diffraction (3D ED) data are collected analogously to SCXRD and SCND using continuous sample rotation, as demonstrated in MicroED and cRED; the latter is implemented in the software Instamatic.^40−43^ Alternatively, 3D ED data can be obtained by combining stepwise rotation with precession or beam tilt,^44,45^ or merging many still diffraction patterns in SerialED.^46^ Recently, the rapid structure determination of organic compounds from nanocrystals was demonstrated using continuous rotation data collection at time scales comparable to SCXRD.^38,39^ During the past years, it has been shown that 3D ED can provide accurate positions of non-hydrogen atoms in MOFs and HOFs.^47,48^ The noncovalent interactions such as hydrogen bonding could be identified based on the donor–acceptor distances. However, 3D ED was mainly used together with PXRD, NMR, and/or density functional theory (DFT) to reveal the entire range of noncovalent interactions in polycrystalline materials.^49−51^ For example, 3D ED was used in combination with NMR to reveal the hydrogen-bonding network in small molecular crystals.^49^ While 3D ED was used to determine the positions of non-hydrogen atoms, NMR was used to then assign correct atom types, locate hydrogen atoms, and derive the protonation state.^49^ Noncovalent interactions involving hydrogen bonding and π-stacking form the basis of heterochiral supramolecular polymerization, which could be resolved using 3D ED data combined with DFT calculations.^51^

Here, we exclusively use 3D ED to determine the structure of a nanocrystalline hybrid organic–aluminophosphate material SCM-34. We show that the entire range of noncovalent interactions involved in material formation can be revealed based on the atomic positions including hydrogens refined against the 3D ED data. The accurate atomic positions provide important insights into the hydrogen-bonding, electrostatic, π–π stacking, and van der Waals interactions between the inorganic and organic components of the hybrid material, as well as the protonation state and the effect of noncovalent interactions on the bond length of the terminal P=O π-bond. We corroborate our results using PXRD and NMR to confirm the unit cell parameters, our structural model obtained from 3D ED data, and the protonation state of the organic and inorganic components.

## Results

We synthesized the hybrid material SCM-34 using 1-(3-aminopropyl)imidazole (API) as the structure-directing agent under hydrothermal conditions (Figure S1, Supporting Methods). The resulting crystals have a plate-like morphology with the average dimensions of 3.0 × 1.5 × 0.2 μm^3^ (Figure S2). cRED data were collected at room temperature from nine crystals during a 2 h session on a JEOL JEM2100 transmission electron microscope (TEM) using the program Instamatic.^43^ The data were processed using XDS,^52^ suggesting a triclinic unit cell (Tables 1, S1, and S2; Figure S3). Seven data sets were selected based on their intensity consistency, which were subsequently merged to achieve an overall completeness of 98.8% up to 0.75 Å resolution (Tables 1 and S3).

**Table 1:** Data Collection and Structure Refinement

crystal data	
formula	|(C_6_N_3_H_13_)_2_|[P_4_Al_2_O_18_H_6_]
crystal system	triclinic
space group	*P*1̅
*a*, *b*, *c* (Å)	6.831, 8.418, 12.068
α, β, γ (deg)	100.78, 101.60, 91.33
*V* (Å^3^)	666.5

data details	
temperature (K)	293
radiation (Å)	electrons, 0.0251
number of crystals	7
*d*_min_, *d*_max_ (Å)	0.75, 11.80
completeness (%)	98.8
total, unique reflection, Rint	18 143, 3258, 0.2507
observed data [*I* > 2.0σ(*I*)]	2385

refinement	
*N*_reflections_, *N*_parameters_, *N*_restraints_	3258, 214, 2
*R*1, w*R*2 [*F*^2^ > 2.0σ(*F*^2^)]	0.1861, 0.4468
*R*1, w**R**2 (all data)	0.2224, 0.4743

**Figure 1:**
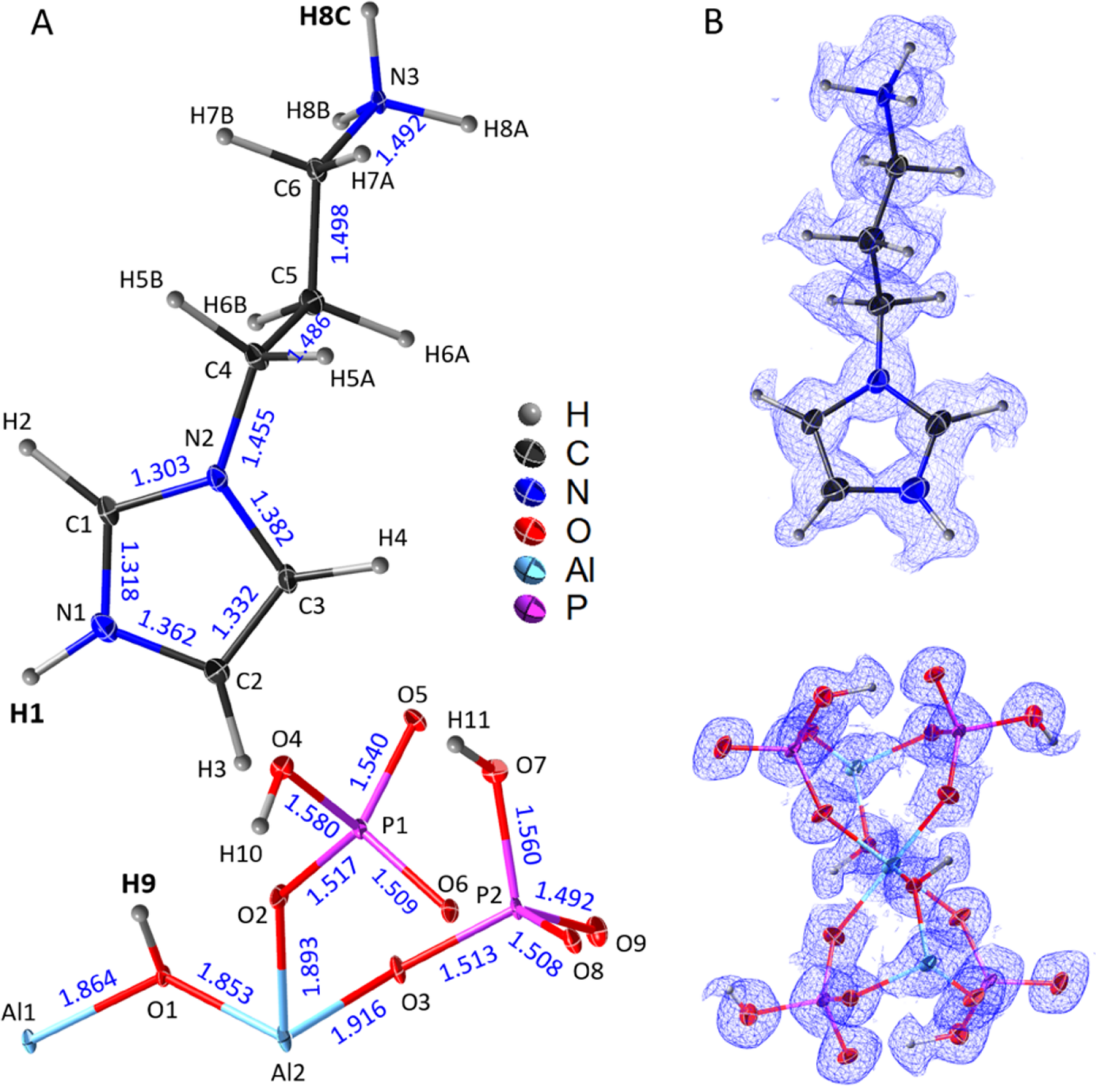
Structure of SCM-34 determined using 3D ED data. (A) The connectivities of 37 atoms in the asymmetric unit of SCM-34 (10% probability displacement ellipsoids). The protonation sites (H1, H8C, and H9, highlighted in bold) are identified in the organic API and the inorganic aluminophosphate chain. Bond lengths between non-hydrogen atoms are indicated (in Å, blue). (B) Observed Fourier map for the API molecule and aluminophosphate chain (isosurface level: 1.35σ).

The structure of SCM-34 was solved ab initio from the merged cRED data using direct methods in SHELXT^53^ in the space group *P*1̅. All non-hydrogen atom (P, Al, C, N, and O) positions were successfully resolved. During structure refinement using SHELXL,^54^ 14 out of 16 symmetry-independent hydrogen atoms were located directly from the strong peaks in difference Fourier maps (Figures 1 and S4). These were then constrained by ideal geometry but allowed to refine the X–H (X = C, N, and O) distances. The exceptions being the C2–H3 and C3–H4 distances that were restrained to the ideal hydrogen bond lengths from a neutron diffraction of 1.08 Å with a σ of 0.02 Å.^55^ The refined chemical composition is |(C_6_N_3_H_13_)_2_[P_4_Al_2_O_18_H_6_], and the refinement converged to *R*1 = 0.186 and w*R*2 = 0.447 (*F*^2^ > 2.0σ(*F*^2^); Table 1).

**Figure 2:**
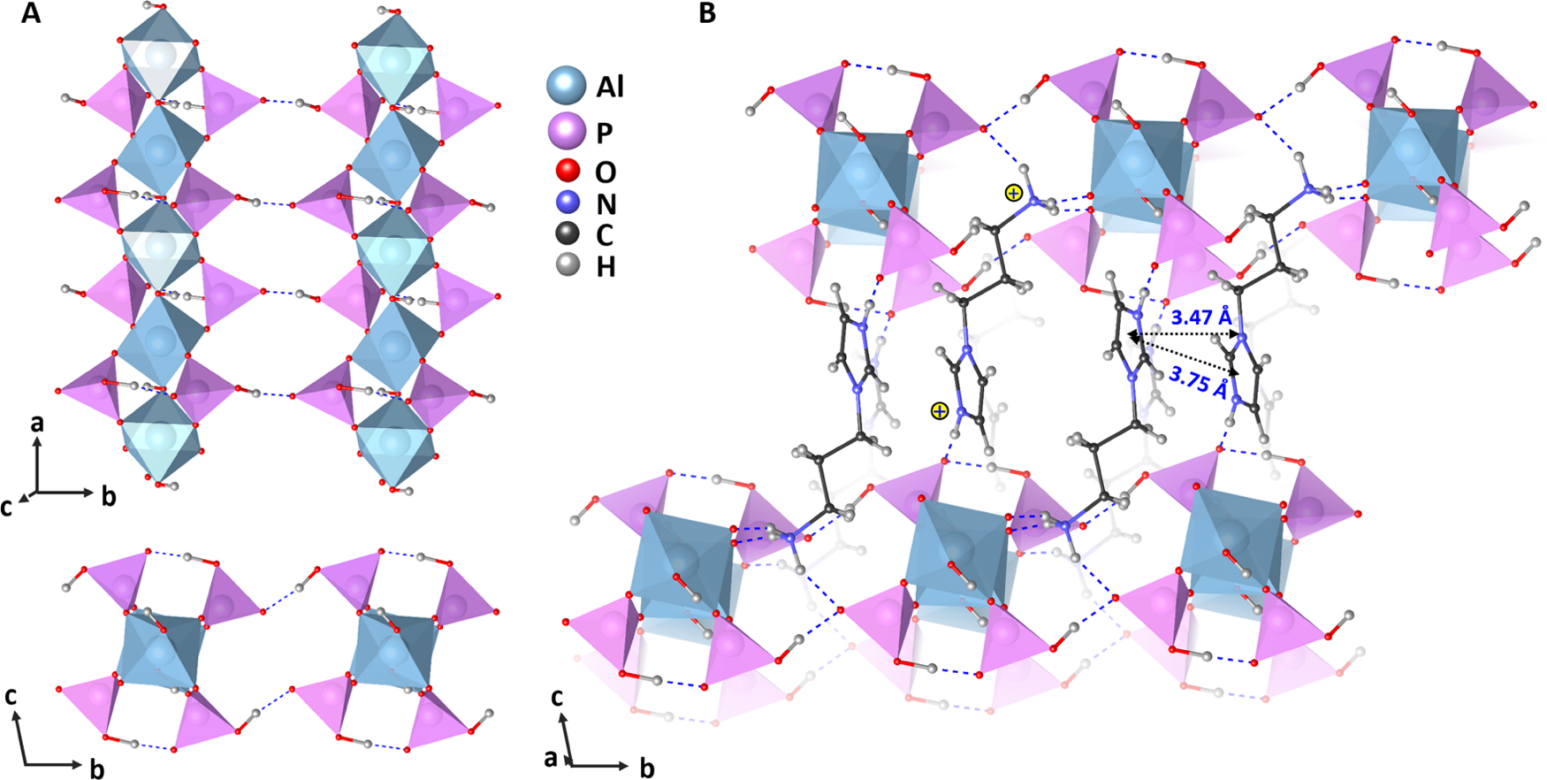
Structural analysis of the resolved hydrogen-bonding, electrostatic, and π–π stacking interactions for SCM-34. (A) Aluminophosphate chains are built from AlO_4_(OH)_2_ octahedrons and O=PO_2_(OH) tetrahedrons. The negatively charged chains are stabilized and aligned together via the hydrogen-bonding interactions (blue dotted line) inside and between the chains. (B) Location of the API molecules and their noncovalent interactions. Each API molecule is double-protonated. The protonated parts (−(NH_3_)^+^, −(NH)^+^−) are approaching the negatively charged aluminophosphate chains and binding the chains along the *b* and *c* directions through the hydrogen-bonding and electrostatic interactions. Two API molecules are packed as a dimer through π–π stacking interactions of imidazole rings.

The SCM-34 structure consists of inorganic aluminophosphate chains interacting with the organic API molecules (Figure 2). The aluminophosphate chains are closely related to those in Na_4_Al(PO_4_)_2_(OH) and AlPO-CJ10.^56,57^ The chains in SCM-34 are built of AlO_4_(OH)_2_ octahedrons (Al^3+^) and O=PO_2_(OH) tetrahedrons (P^5+^), arranged along the crystallographic *a* direction. The adjacent AlO_4_(OH)_2_ octahedrons are connected via shearing the protonated O1 atoms (proton: H9) and are further bridged by the O=PO_2_(OH) tetrahedrons (Figures 1 and 2). Two protons (H1 and H8C) were identified in each API molecule, indicating that each API molecule was double-protonated during the synthesis (Figure 1). In the chains, the bond lengths of Al–O bonds in Al–O–Al and Al–O–P are 1.853(6)–1.864(4) Å and 1.885(7)–1.930(6) Å, respectively (Table S4), while the bond lengths between P and O in P–O–H, P–O–Al, and P=O are 1.560(9)–1.580(8) Å, 1.508(7)–1.517(6) Å, and 1.492(8)–1.540(7) Å, respectively (Table S4). In the API molecule, the C–N bond lengths in the imidazole ring and the chain tail are 1.318(14)–1.382(15) and 1.455(12)–1.492(10) Å, respectively (Table S5). The C–C bond length in the imidazole ring is 1.332(13) Å, much shorter than those in the chain tail (1.486(13)–1.498(13) Å).

**Figure 3:**
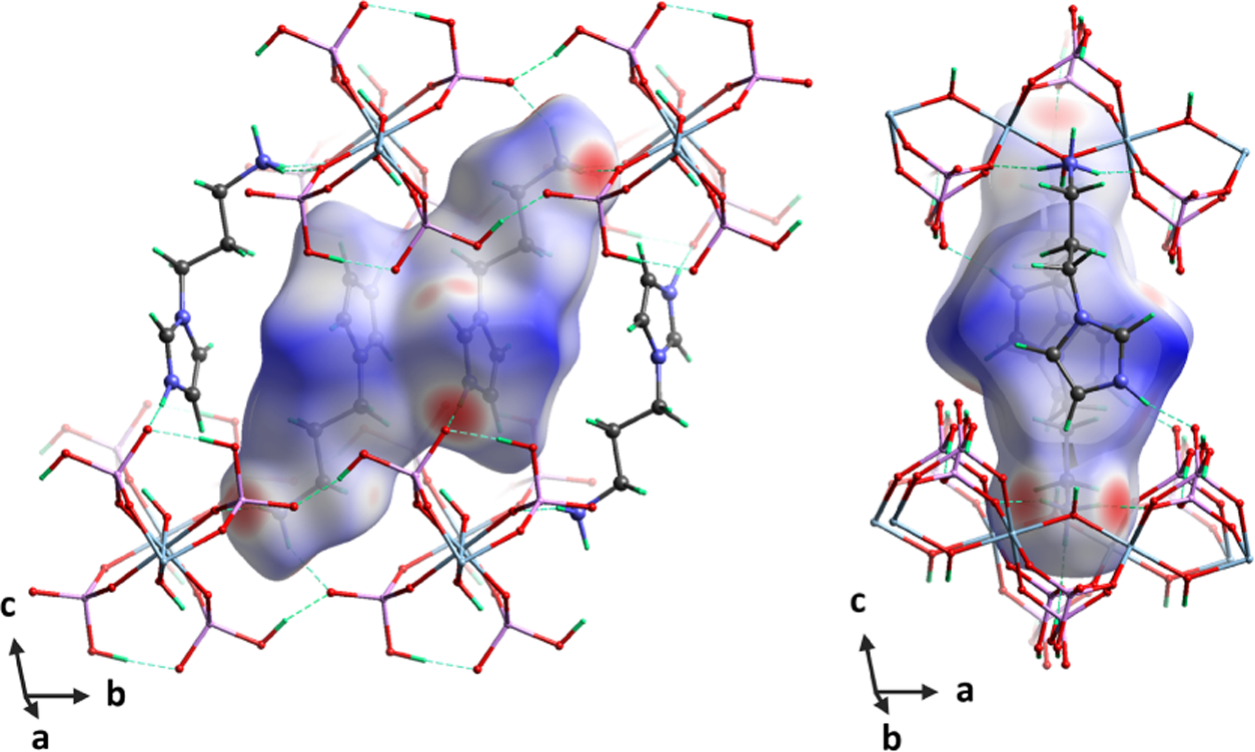
Hirshfeld surface for the API molecules (mapped with *d*_norm_ over the range of −0.806–1.932) in SCM-34. The color scheme used on this surface indicates the contact distance to the aluminophosphate chains: contacts that are shorter than the sum of the van der Waals radii are colored red, contacts equal to the sum of the van der Waals radii are colored white, and blue represents the longer contacts.^59^

The hybrid structure of SCM-34 is assembled from the aluminophosphate chains and API molecules through different types of noncovalent interactions (Figure 2). In the aluminophosphate chains, the neighboring O=PO_2_(OH) tetrahedrons interact via hydrogen bonds between their terminal P–OH and O=P groups. The distance between the donor (D) and acceptor (A) atoms (P–O7–H11···O5=P) is 2.554(10) Å, indicating that a strong interaction is formed to stabilize the chains. Meanwhile, the aluminophosphate chains are further aligned via strong hydrogen-bonding interactions (P–O4–H10···O9=P) between their neighboring parallel chains (Figure 2A and Table 2). The summed composition of the chains ([P_4_Al_2_O_18_H_6_]^4–^) within a single unit cell has a negative charge of −4, which is balanced by the positive charge from the two double-protonated API molecules (|(C_6_N_3_H_13_)_2_|^4+^). The protonated parts (−(NH_3_)^+^, −(NH)^+^−) of the API molecules are approaching the negatively charged chains, binding to the chains along the *b* and *c* axes through hydrogen-bonding and electrostatic interactions (Figure 2B). All hydrogen bonds are chemically reasonable, and their strengths were deduced from the distances of H···A and D···A and the angles of D–H···A (Table 2 and Figure 2).^30,31^ The −(NH_3_)^+^ part interacts via three moderately strong hydrogen bonds (N3–H8A···O8, N3–H8B···O9, and N3–H8A···O3) to the aluminophosphate chain. The −(NH)^+^– part has a strong hydrogen-bonding interaction (N1–H1···O5). The API molecules are observed to be packed as dimers via the imidazole rings with the shortest distance of 3.47 Å and a central distance of 3.75 Å, indicating the formation of the offset-type π–π stacking interaction.^58^ In addition, the van der Waals interactions can be visualized by the Hirshfeld surface for the structure (Figure 3).

**Table 2:** Hydrogen-Bonding Interactions in SCM-34

donor–H···acceptor	D–H (Å)	H···A (Å)	D···A (Å)	D–H···A (°)	interaction strength	details of the acceptor groups
N1–H1···O5	1.01(5)	1.57(5)	2.586(10)	167(5)	strong	P=O terminal O
N3–H8A···O8	1.04(4)	1.79(4)	2.806(9)	164(3)	moderate	P–O–Al bridge O
N3–H8B···O9	1.04(4)	1.72(4)	2.688(12)	151.5(11)	moderate	P=O terminal O
N3–H8C···O3	1.04(4)	1.77(4)	2.781(8)	162(2)	moderate	P–O–Al bridge O
O4–H10···O9	0.97(5)	1.56(5)	2.505(9)	165(5)	strong	P=O terminal O
O7–H11···O5	1.08(6)	1.48(6)	2.554(10)	171(5)	strong	P=O terminal O

To corroborate our results, we performed additional characterizations that were only used for structure validation. The unit cell dimensions derived from the PXRD data are in good agreement with those determined from our cRED data (Tables 1 and S2; Figure S1). The maximum deviations of the unit cell lengths and angles are 0.22 Å and 0.14°, respectively. The structural model of SCM-34 was validated using ^31^P, ^27^Al, and ^13^C NMR spectroscopy, inductively coupled plasma (ICP), and chemical element analysis. The ^31^P and ^27^Al NMR spectra indicate that P and Al are four- and six-coordinated, respectively (Figure S5). The ^13^C NMR spectrum shows that the API molecules are accommodated in the structure and could be double-protonated (Figure S6). The calculated molar ratios of P/Al and C/N in SCM-34 are 2.1 and 2.0, respectively, which is consistent with their molar ratios in the chemical composition (|(C_6_N_3_H_13_)_2_|[P_4_Al_2_O_18_H_6_]) resolved from the cRED data. ^1^H solid-state NMR and Fourier transform infrared (FT-IR) spectroscopies were applied to detect the protonation state of the API molecules and aluminophosphate chains. The ^1^H solid-state NMR spectrum shows a broad peak centered at 6.78 ppm and a sharp peak at 0.86 ppm (Figure S7). The sharp peak at 0.86 ppm was assigned to Al–OH or P–OH groups;^60^ the broad peak (6.78 ppm), however, could not offer any information regarding the protonation state of the API molecules. In the FT-IR spectrum, the signal attributed to Al–OH and P–OH groups is overlapping at 3657 cm^–1^, and the signal of different C–H and N–H groups is overlapping in the region of 2750–3200 cm^–1^ (Figure S8).^61^ The signal overlapping in the ^1^H solid-state NMR and FT-IR spectra makes it challenging to reliably interpret the protonation state of the API molecules and the aluminophosphate chains without accurate structure information. The thermal stability of SCM-34 was studied by ex situ PXRD, in situ electron diffraction, and thermogravimetric analysis–differential scanning calorimetry (TGA–DSC). The ex situ PXRD experiments show that the structure was stable up to 150 °C (Figure S9). In situ electron diffraction experiments show that the structure of SCM-34 was stable up to 100 °C and collapsed at 150 °C in vacuum (Figure S10). TGA–DSC analysis indicates that the structure collapsed before 185 °C (Figure S11).

## Discussion and Conclusions

We present the structure determination of the nanocrystalline hybrid material SCM-34. All atomic positions were resolved from our 3D ED data, including the hydrogen atoms (Figure 1). The X–H (X = C, N, O) hydrogen bond lengths, except those of C2–H3 and C3–H4, were refined without restraints and are on average longer than the idealized hydrogen bond lengths from X-ray diffraction (averaged deviation: 0.164 Å; Table S6).^55^ This is in line with previous observations that the hydrogen bond lengths observed in electron diffraction are closer to the inter-nuclei distances observed by neutron diffraction.^40,62^ The bond lengths of C2–H3 and C3–H4 had to be restrained as the positions of H3 and H4 atoms were poorly resolved in our electrostatic potential map (Figures 1 and S4). This may be because H3 and H4 are located in a region of the API molecule that has higher structural flexibility and due to the fact that the two hydrogen atoms likely are more disordered as they are facing outward the empty pockets between the chains (Figure 2). The hydrogen atoms involved in noncovalent hydrogen-bonding interactions between the aluminophosphate chains and API molecules were unambiguously identified from strong difference peaks in the map (Figure S4). The protons H1, H8C, and H9 in the structure indicate that the aluminophosphate chains and the API molecules were protonated during the synthesis.

The covalent bond lengths between the non-hydrogen atoms in our structure are accurate with an average deviation of 0.013 Å compared to the reported SCXRD bond lengths, enabling the assignment of each bonding type (Tables S4 and S5). In the API molecules, the bond lengths of N1–C1, N1–C2, N2–C4, N3–C6, C2–C3, and C5–C6 are almost identical to their corresponding reported SCXRD bond lengths (0.003 Å deviation). The C–N and C–C bonds in the imidazole ring and the tail of the API molecule can be distinguished based on their bond lengths (Table S5). In the aluminophosphate chains, based on the observed bond lengths, we can distinguish Al–O bonds in Al–O–Al (1.853(6)–1.864(4) Å) and Al–O–P (1.885(7)–1.930(6) Å) (Table S4). The different bond lengths between P and O in P–O–H, P–O–Al, and P=O can be identified from our data, with the exception of the P1=O5 terminal bond length (1.540(7) Å; Table S4). Notably, the bond length of P1=O5 (1.540(7) Å) is significantly longer compared to the expected bond length (1.500 Å) and the P2=O9 (1.492(8) Å) bond length. This elongation may be the result of strong hydrogen-bonding and electrostatic interactions with this oxygen atom (*d*_O5···O7_ = 2.554(10) Å in P1=O5···H11–O7 and *d*_O5···N1_ = 2.586(10) Å in P=O5···H1–N1^+^). Systematic studies have shown the influence of strong hydrogen-bonding interactions on the lengthening of terminal C=O bonds.^28^ The electron of the P1=O5 π-bond can be delocalized by the hydrogen-bonding interaction, and the formal charge of the O5 atom accordingly becomes −1.^63,64^ The strong electrostatic interactions between O5 and N1^+^ therefore may contribute to elongation of the terminal bond P1=O5. The shorter bond length of P2=O9 may be the result of weaker hydrogen-bonding and electrostatic interactions (Table 2 and Figure S12).

The hydrogen bond interactions were resolved based on the identified positions of the hydrogen, donor, and acceptor atoms in the structure. The strength of each interaction was interpreted based on the distances of H···A and D···A and the angles of D–H···A (Table 2).^65^ The determined protons (H1, H8C, and H9) demonstrate the electrostatic interactions between the positively charged API molecules and the negatively charged aluminophosphate chains. The API molecules are packed as dimers via offset-type π–π stacking interactions between the imidazole rings. Furthermore, van der Waals interactions between the chains and API molecules can be visualized from the Hirshfeld surface.^59^ These noncovalent interactions can be calculated based on the accurate structural model, which enables the quantitative analysis of noncovalent interactions.^66^

Identifying the entire range of noncovalent interactions in SCM-34 enables us to deduce the formation mechanism of this organic and inorganic hybrid material. Each aluminophosphate chain is built of AlO_4_(OH)_2_ octahedrons and O=PO_2_(OH) tetrahedrons that are stabilized via hydrogen-bonding interactions inside the chain. The resulting chains are further aligned with the hydrogen-bonding interactions between their neighboring parallel chains. To extend the structure into three dimensions along the *b* and *c* directions, API molecules are double-protonated and packed as dimers via π–π stacking interactions to place the hydrogen-bonding and electrostatic interactions with the parallel aligned aluminophosphate chains and build the organic–inorganic hybrid system. Van der Waals interactions play a role in shaping and supporting the hybrid structure.

The structure of the aluminophosphate chain in SCM-34 is similar to those reported previously in hybrid aluminophosphate materials, which were all determined by SCXRD from much larger crystals.^56,57,67,68^ Being able to determine the detailed structures of nanocrystalline materials, 3D ED can complement SCXRD and reveal the entire range of noncovalent interactions from nanocrystals. This method can also be applied for studying noncovalent interactions in other hybrid and nonhybrid materials such as hydrogen-bonded organic frameworks (HOFs) and covalent organic frameworks (COFs).^48,69^ We anticipate 3D ED to provide new insights into the formation mechanism of crystalline materials constructed via noncovalent interactions to improve our understanding of supramolecular chemistry in polycrystalline materials and facilitate the development of novel functional materials.

## Supporting Information Available

Synthetic procedures, characterization methods and data (3D ED, XRD, SEM, TGA, NMR, etc.), and structure refinement details of SCM-34 (PDF)

## Accession Codes

CCDC 2149680 contains the supplementary crystallographic data for this paper. These data can be obtained free of charge via www.ccdc.cam.ac.uk/data_request/cif, or by emailing data_request@ccdc.cam.ac.uk, or by contacting The Cambridge Crystallographic Data Centre, 12 Union Road, Cambridge CB2 1EZ, UK; fax: +44 1223 336033.
