# Supplementary material for: Visualizing the Entire Range of Noncovalent Interactions in Nanocrystalline Hybrid Materials Using 3D Electron Diffraction
Source: J Am Chem Soc. 2022 Jun 9;144(24):10817–24. doi: 10.1021/jacs.2c02426 (PMC9490833; doi:10.1021/jacs.2c02426)
Supplement: Supplementary file 1 — ja2c02426_si_001.pdf [file ja2c02426_si_001.pdf]

Supplementary materials for

Visualizing the entire range of noncovalent interactions in nanocrystalline hybrid materials using 3D electron diffraction

Yi Luo[1,†,*], Max T.B. Clabbers[1,†], Jian Qiao[2], Zhiqing Yuan[2], Weiming Yang[2,*], and Xiaodong Zou[1,*]

[1]Department of Materials and Environmental Chemistry, Stockholm University, SE-106 91 Stockholm, Sweden.

[2]State Key Laboratory of Green Chemical Engineering and Industrial Catalysis, Sinopec Shanghai Research Institute of Petrochemical Technology, 1658 Pudong Beilu, Shanghai 201208, China.

[*]Correspondence authors: X.Z. (xzou@mmk.su.se), W.Y. (yangwm.sshy@sinopec.com), and Y.L. (yi.luo@mmk.su.se)

[†]These authors contributed equally to this work.

Table of contents

Methods……………………………………………………………………………..………………3
Figure S1…………………………………………………………………………………………4
Figure S2…………………………………………………………………………………………5
Figure S3…………………………………………………………………………………………6
Figure S4…………………………………………………………………………………………7
Figure S5…………………………………………………………………………………………8
Figure S6…………………………………………………………………………………………9
Figure S7……………………………………………………………………………………...…10
Figure S8……………………………………………………………………………………...11
Figure S9……………………………………………………………………………………...12
Figure S10..……………………………………………………………………………………...13
Figure S11..……………………………………………………………………………………...14
Figure S12..……………………………………………………………………………………...15
Table S1………………………………………………………………………….……...16
Table S2………………………………………………………………………….……...17
Table S3………………………………………………………………………….……...18
Table S4………………………………………………………………………….……...19
Table S5………………………………………………………………………….……...20
Table S6………………………………………………………………………….……...21
References…………………………………………………………………………….………22

Methods

Synthesis. SCM-34 was synthesized using 1-(3-Aminopropyl)imidazole (API, $C_6N_3H_{11}$, 97 wt%, Sigma Aldrich) as a structure-directing agent under hydrothermal conditions. In the typical synthesis procedure, 1.09 g aluminum isopropoxide ($Al(iPr)_3$, 98 wt%, Sinopharm Chemical Reagent Co., Ltd) was added into 4 g distilled water and the mixture was then stirred at room temperature for 30 min. Afterward, 2.5 g phosphoric acid (H_3PO_4, 85 wt%, Sinopharm Chemical Reagent Co., Ltd) and 4 g distilled water were introduced, and the mixture was stirred for 60 min. Later, 1.89 g API liquid was added per drop. In the end, 0.015 g tetraethylammonium hydroxide solution (TEAOH, 25 wt%, Sinopharm Chemical Reagent Co., Ltd) was added and stirred for 30 min. The molar composition of the obtained synthesis gel is 1 P_2O_5 : 0.25 Al_2O_3 : 1.5 $C_6N_3H_{11}$: 0.004 TEAOH : 43H_2O. The resulting gel was transferred into a Teflon-lines steel autoclave, aged at 60 ºC for 12 h, and then heated at 140 ºC for 72h. The solids were recovered by centrifugation, washing, and drying (110 ºC, 12h).

3D ED data collection and structure determination. 3D electron diffraction data were collected using the continuous rotation method MicroED[1] implemented as cRED in the software *Instamatic*[2], on a JEOL JEM2100 (LaB6 filament) transmission electron microscope (TEM) operated at 200 kV, equipped with a Timepix hybrid pixel detector (Amsterdam Scientific Instruments). Data were collected using a high tilt side-entry holder (JEOL) at room temperature. The reflection intensities were integrated, scaled, and merged using *XDS*[3]. The structure was solved using direct methods in *SHELXT*[4] and refined using *SHELXL*[5] and Shelxle[6]. Electron atomic scattering factors (eight parameter fitting) were used. The command SWAT was used considering disordered water molecules in the structure.

In-situ electron diffraction. In-situ heating experiments were performed on a FEI Themis Z aberration-corrected TEM using a heating sample holder (Gatan, model 625). The heating rate was 10 °C/min. The temperature was held for 30 min at each test temperature before the selected area electron diffraction pattern was collected (exposure time 0.64 s). The crystal was only illuminated by electron beam when electron diffraction patterns were recorded.

Other characterizations. Powder X-ray diffraction (PXRD) patterns were collected on a PANalytical X'Pert PRO diffractometer with Cu $K\alpha$ radiation, operated at 40 kV and 40 mA in the 2θ range of 5°-40°. Scanning electron microscopy (SEM) measurements were performed on a Hitachi S-4800 field-emission scanning electron microscope (Hitachi, Japan) with an acceleration voltage of 3 kV. Thermogravimetric analysis (TGA) and Differential Scanning Calorimetry were conducted on a TA SDT-Q600 instrument by heating the samples up to 800 °C at a rate of 10 °C/min. Inductively coupled plasma-atomic emission spectrometry (ICP-AES) measurements were performed using a Thermo IRIS Intrepid II XSP atomic emission spectrometer after dissolving the samples in HF solution. FTIR spectrum was recorded using a Thermo Fisher Nicolet 380 spectrometer. Elemental analyses of C, N, and H were conducted on an ElementarVario MICRO CUBE

elemental analyzer. ^{31}P, ^{27}Al, ^{13}C, and ^{1}H magic angle spinning nuclear magnetic resonance (MAS NMR) measurements were performed using a Varian-400 spectrometer.

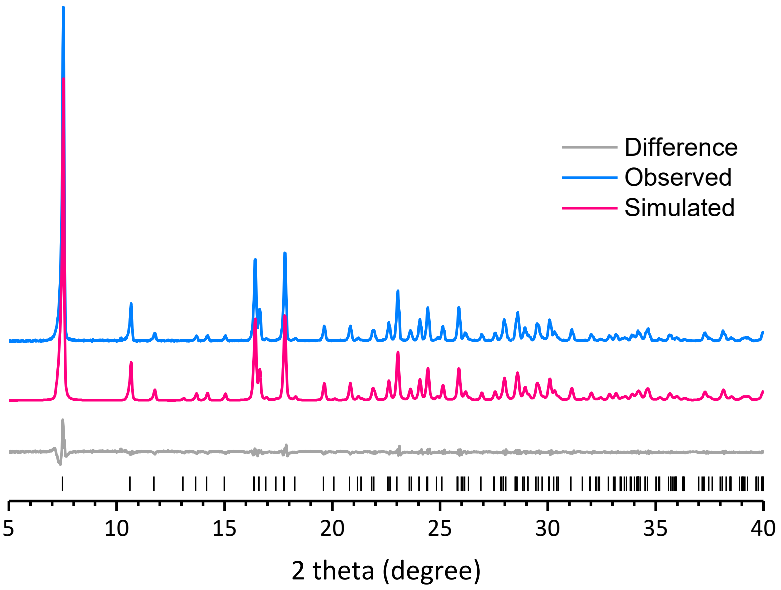

Figure S1. PXRD pattern (Cu kα) of SCM-34 and its Pawley fit results. Observed (blue line), calculated (pink line), and difference profiles (grey line) are presented. The black tick marks under the profiles are the positions of the Bragg reflections. The crystal has a triclinic unit cell of a = 6.9181 Å, b = 8.4938 Å, c = 12.2885 Å, α = 100.923°, β = 101.503°, and γ = 91.256°.

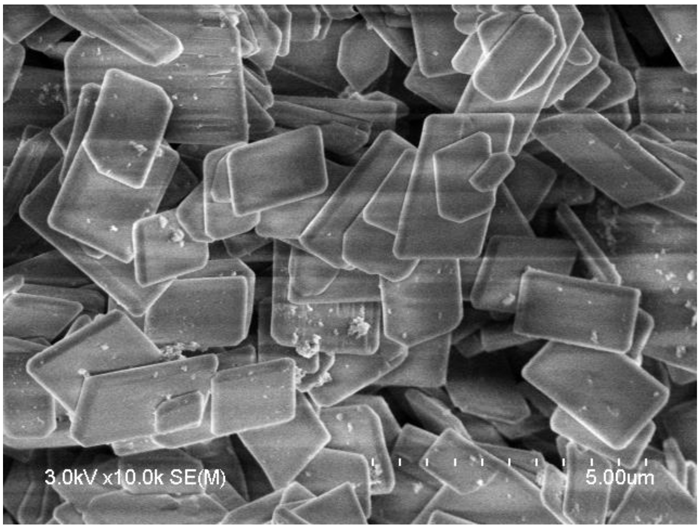

Figure S2. SEM image of SCM-34. Crystals show a typical plate-like morphology with average dimensions of about 3.0×1.5×0.2 μm^3.

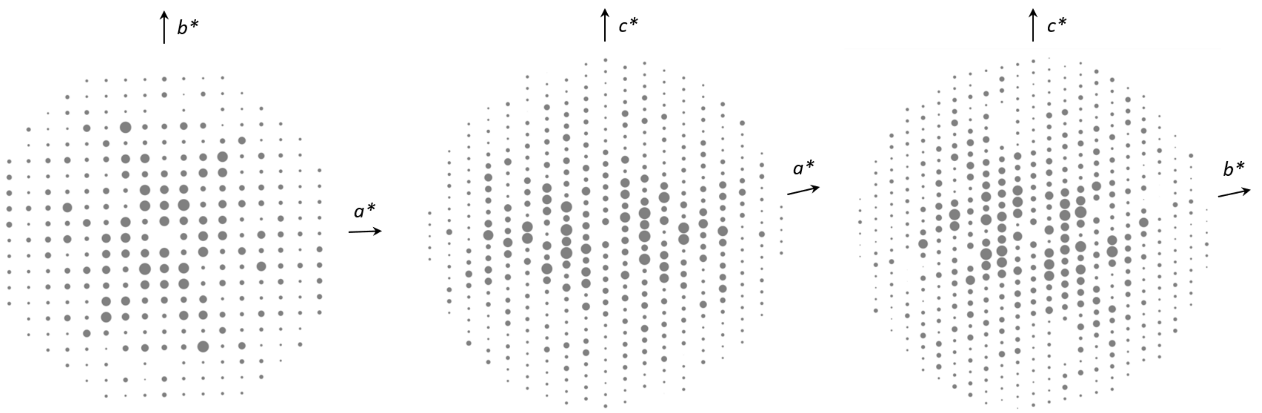

Figure S3. 3D reciprocal lattice of SCM-34 reconstructed from the merged cRED data, viewed along the *c*-, *b*- and *a*- axes. The edges of the circles correspond to 0.75Å resolution.

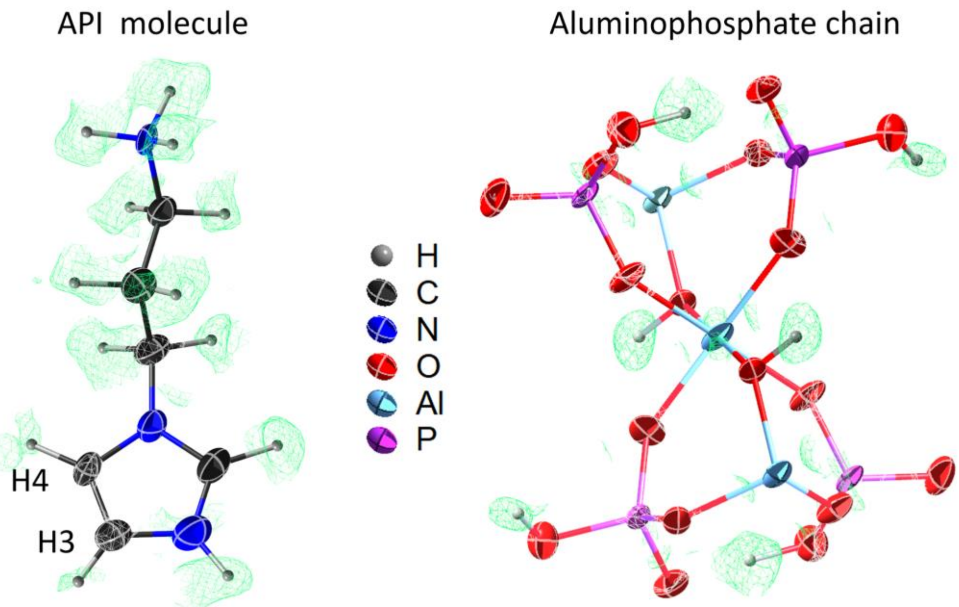

Figure S4 Difference Fourier map (green) showing hydrogen atoms in API molecules and aluminophosphate chains (isosurface level: 1.40σ). The map is generated by omitting the hydrogen atoms.

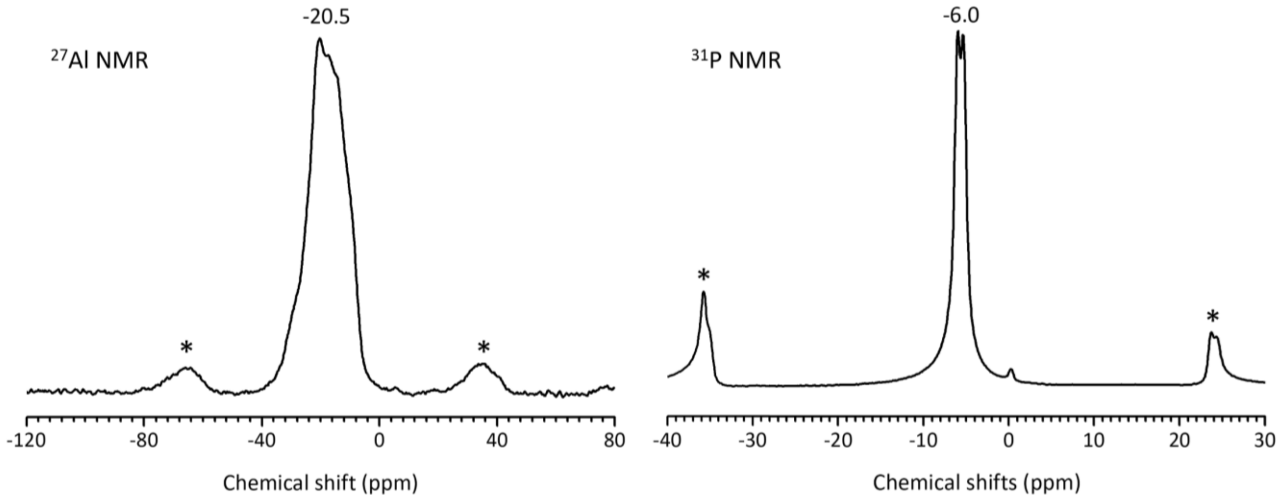

Figure S5. ^{27}Al and ^{31}P solid-state NMR spectra of SCM-34. The chemical shift centered at -20.5 ppm in the ^{27}Al NMR spectrum and the chemical shift centered at -6 ppm in the ^{31}P NMR spectrum indicate the Al and P atoms in the structure of SCM-34 are 6-coordinated and 4-coordinated, respectively.[7,8] Peaks marked with asterisks are spin sidebands.

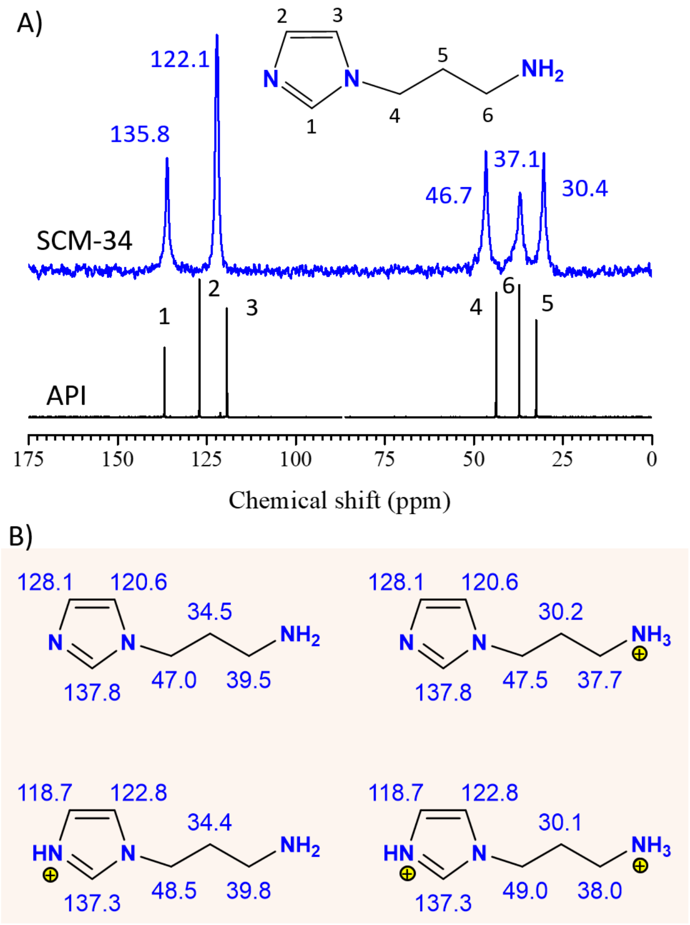

Figure S6. A) ^{13}C liquid NMR spectrum of the API molecule and the ^{13}C solid-state NMR spectrum of SCM-34. B) The simulated ^{13}C NMR chemical shifts (blue numbers, ppm) of API molecules with different protonation states (simulated by ChemDraw).

The spectra of SCM-34 indicate that the API molecules are accommodated in the structure. C2 and C3 seem to have a very similar chemical environment in SCM-34, as there is only one peak showing up in between their chemical shifts in the ^{13}C liquid NMR spectrum. This indicates the imidazole ring could be protonated. The decreasing of the chemical shift of C5 indicates the N atom connected to the C6 could be protonated. These can be indirectly deduced by the simulated ^{13}C NMR spectra of API molecules with different protonation states. However, the complex interactions and chemical environment in SCM-34 could also affect the chemical shifts of some C atoms, which could make the interpretation ambiguous.

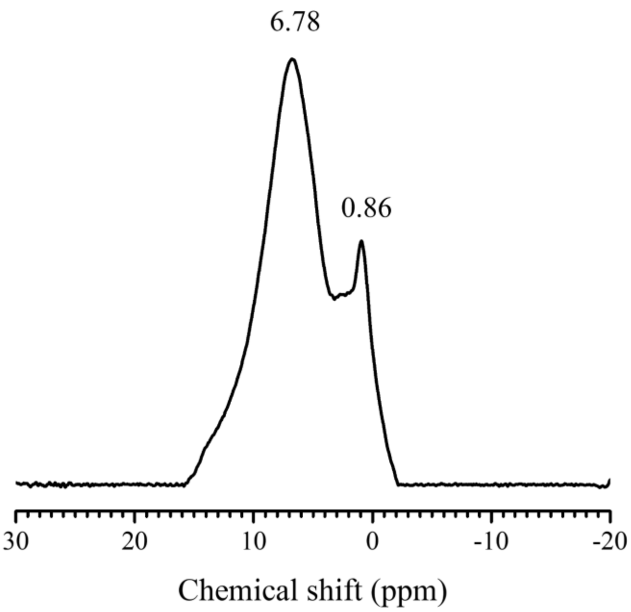

Figure S7. ^{1}H solid-state NMR spectrum of SCM-34.

The chemical shifts of H atoms connected to the imidazole ring of the API molecule are in the 6.5-8.0 ppm region. While the chemical shits of H atoms connected to the tail of the API molecule should be in the region of 1.5-4.5 ppm.[9] In the spectrum, the sharp peak at 0.86 ppm can be attributed to Al–OH or P–OH groups,[10] while the broad peak centered at 6.78 ppm could not offer any useful information regarding the protonation of API molecule.

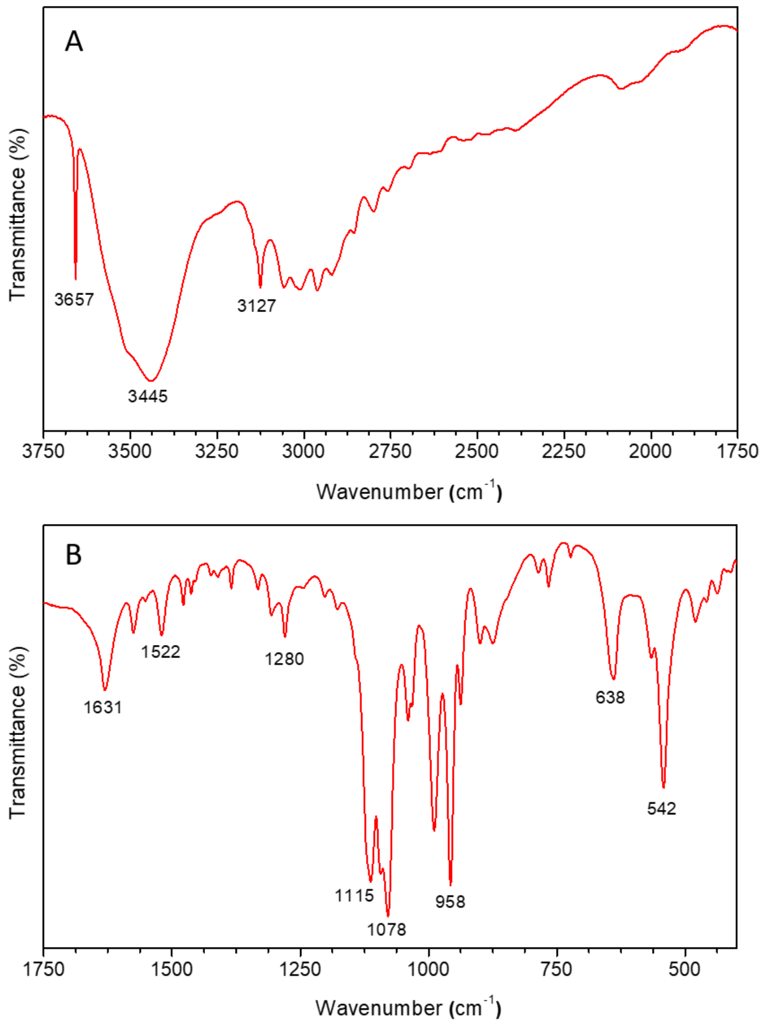

Figure S8. FT-IR spectrum of SCM-34 in regions 1750-3750 cm^{-1} (A) and 400-1750 cm^{-1} (B). Absorption below 1400 cm^{-1} is attributed to the lattice vibration of aluminophosphates. Peaks at 950-1250 and 600-900 cm^{-1} regions correspond to the asymmetric and symmetric stretching vibrations of AlO_6 octahedrons and PO_4 tetrahedrons. The 400-600 cm^{-1} are due to O–T–O (T=Al, P) bending. The API molecule gives absorptions in the regions 2750-3200 and 1400-1700 cm^{-1}, which could be attributed to X–H (X=C and N) stretching and bending vibrations. The region 3250-3700 cm^{-1} can be attributed to the stretching vibrations of OH groups. As presented in the spectrum, there is only one sharp peak at 3657 cm^{-1}, which can be attributed to P–OH and/or Al–OH–Al groups. Therefore, without the accurate structure model resolved by 3D ED, it is almost impossible to distinguish the different types of OH groups due to the peak overlapping.[7,9,11]

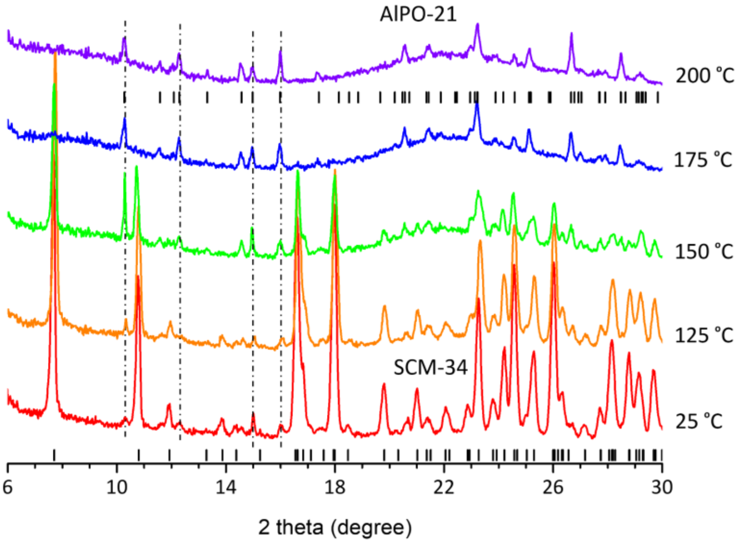

Figure S9 PXRD patterns of a SCM-34 sample heated at 125, 150, 175 and 200 °C, respectively, with a heating rate of 5 °C/min. The sample was kept at each given temperature for 30 min and then cooled down to room temperature for the PXRD data collection. The experiments were conducted on the same sample at the four given temperatures. The ex-situ PXRD experiments show that SCM-34 was stable up to 150 °C. This sample contains a small amount of impurity AlPO-21 ($P2_1/n$, a= 8.676 Å, b=17.456 Å, c=9.156 Å, β=109.7°).[12] The black tick marks under the red and purple profiles correspond to the peak positions of SCM-34 and AlPO-24, respectively.

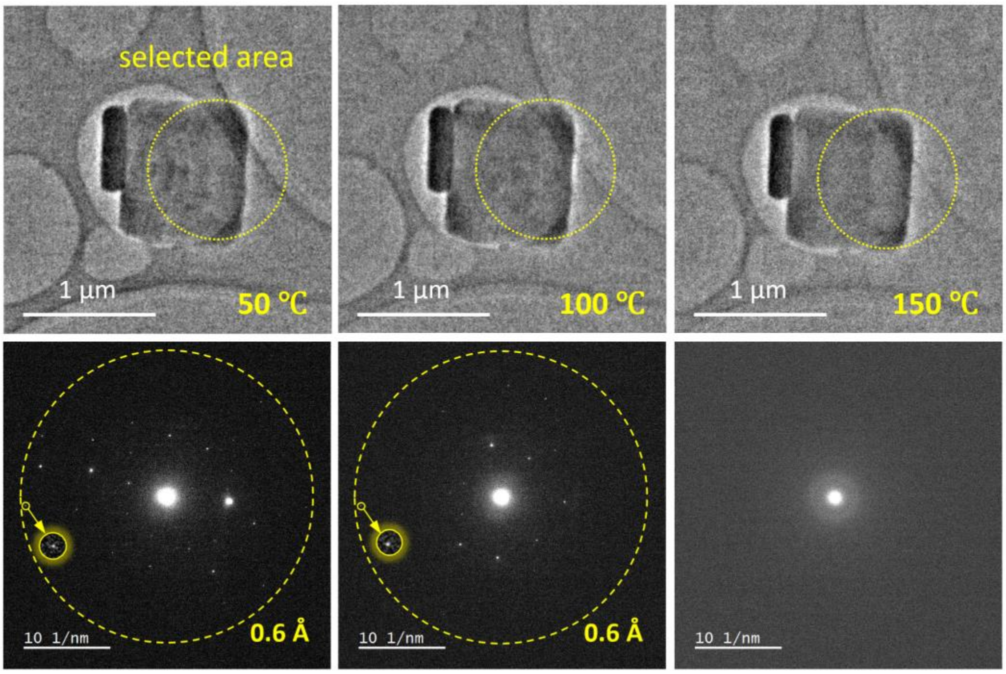

Figure S10 In-situ heating electron diffraction experiments (vacuum ~1×10^{-5} Pa) on SCM-34. The TEM images (top row) and the corresponding electron diffraction patterns (bottom row) were acquired on a crystal that was heated at 50 °C, 100 °C, and 150 °C, respectively. The electron diffraction patterns show that the structure of the SCM-34 crystal was stable at 100 °C under vacuum and diffracted to 0.62 Å resolution, but collapsed before 150 °C. The intensity differences in the ED patterns at 50 °C and 100 °C can be attributed to a slight change of the crystal orientation during the heating.

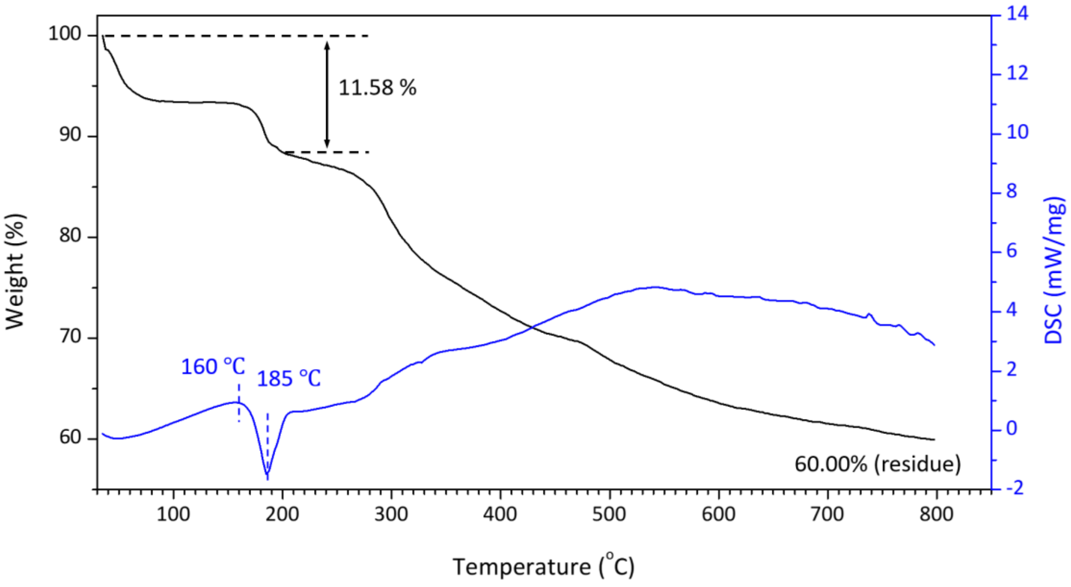

Figure S11. TGA-DSC analysis of SCM-34. The TGA curve shows a significant weight loss (11.58%) before 200 °C, which can be attributed to the adsorbed water. The DSC curve shows a strong endothermic response starting from ~160 °C with its maximum at 185 °C, which correlates to the collapse of the SCM-34 structure. The weight loss from ca 200 °C was then assigned to the species released from the collapsed SCM-34.

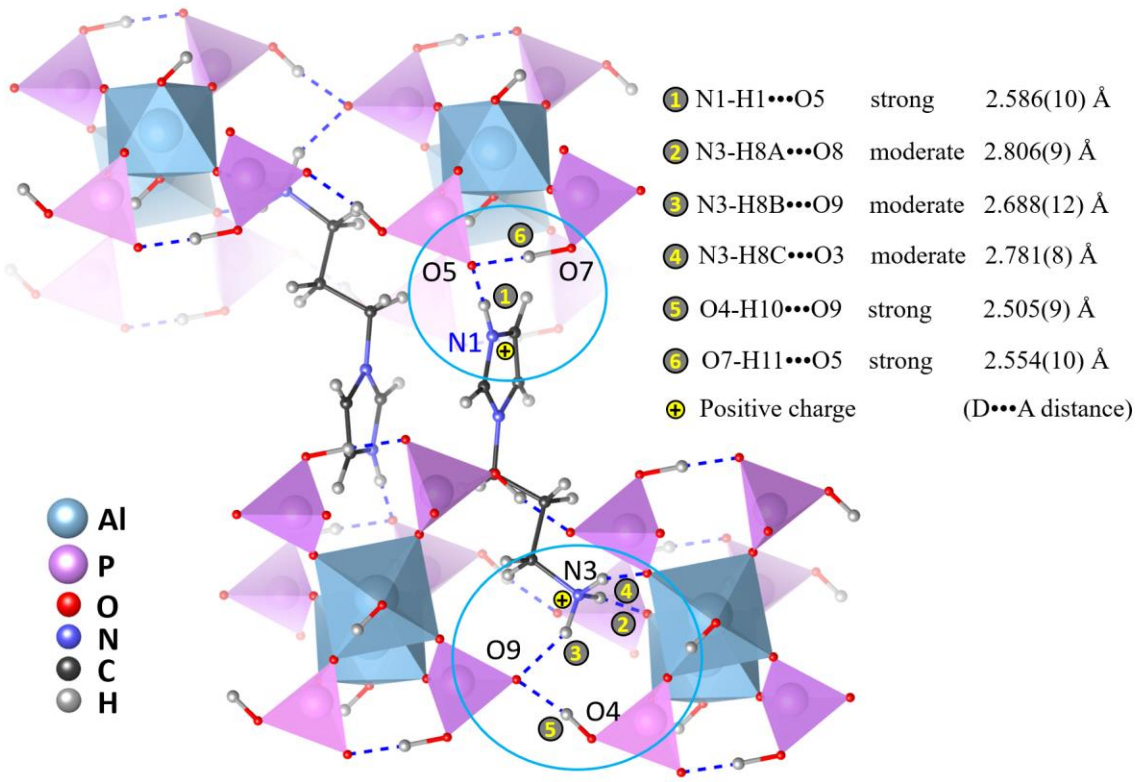

Figure S12 Comparison of the hydrogen bonding and electrostatic interactions on the P1=O5 and P2=O9 terminals. The P1=O5 terminal interacts via two strong hydrogen bonds (N1–H1•••O5 and O7–H11•••O5), while the P2=O9 terminal interacts via one strong (O4–H10•••O9) and one medium strength (N3–H8B•••O9) hydrogen bond. Meanwhile, the electrostatic interaction for O5 ($N1^+$, distance: 2.568(10) Å) would also be stronger than that of O9 ($N3^+$, distance: 2.688(12) Å) due to the shorter distance and higher charge density ($N1^+$ only has one electrostatic interaction with O5, while $N3^+$ has electrostatic interactions with O9, O3, and O8).

Table S1. Data collection parameters of individual crystal datasets.

Dataset	Angular increment (°)	Exposure time (s^{-1})	Number of frames	Rotation range (°)	Detector distance (mm)
1	0.12	0.25	610	79.86	262
2	0.14	0.30	675	102.30	262
3	0.23	0.5	372	94.78	262
4	0.19	0.4	537	111.29	262
5	0.19	0.4	495	101.74	262
6	0.19	0.4	494	101.14	262
7	0.19	0.4	540	111.65	262

Table S2. Unit cell parameters of individual crystal datasets determined by XDS[3].

Dataset	a (Å)	b (Å)	c (Å)	α (°)	β (°)	γ (°)
1	6.837	8.417	12.068	101.292	101.669	91.412
2	6.825	8.418	12.053	100.895	101.697	91.070
3	6.829	8.435	12.106	100.765	101.467	91.344
4	6.820	8.378	12.062	100.901	101.477	91.219
5	6.818	8.414	12.060	100.696	101.498	91.314
6	6.842	8.436	12.080	100.036	101.914	91.386
7	6.846	8.426	12.045	100.850	101.494	91.534

Table S3. Data merging statistics

Resolution limit	Observed reflections	Unique reflections	Complete-ness (%)	R_{merge} (%)	R_{meas} (%)	$I/\sigma I$	$CC_{1/2}$
3.35	167	37	97.4	21.8	24.7	6.07	90.8
2.37	368	66	101.5	19.1	20.9	6.94	93.0
1.94	488	93	96.9	20.8	22.8	6.32	94.3
1.68	493	94	96.9	21.0	23.1	6.34	95.7
1.50	667	121	101.7	23.7	25.9	6.01	96.0
1.37	625	120	100.0	24.3	26.9	5.49	95.1
1.27	838	155	98.1	24.6	27.1	5.41	94.0
1.19	771	143	100.0	23.5	25.9	5.40	96.7
1.12	853	163	98.2	30.6	33.9	4.79	84.2
1.06	927	170	100.0	28.3	31.3	4.83	91.7
1.01	928	173	98.9	34.6	38.2	4.37	90.3
0.97	932	181	98.4	33.1	37.0	3.76	89.6
0.93	1150	197	98.0	37.4	41.3	3.73	89.4
0.90	1125	204	101.5	41.2	45.3	3.60	84.7
0.87	1153	208	98.1	47.5	52.2	3.16	77.1
0.84	1204	207	97.6	43.5	47.7	3.07	76.4
0.81	1386	238	98.3	41.3	45.4	3.20	74.2
0.79	1322	224	98.7	43.0	47.0	3.15	87.7
0.77	1300	224	100.0	51.4	56.3	2.60	72.6
0.75	1446	240	96.8	54.3	59.5	2.33	74.5
total	18143	3258	98.8	26.5	29.2	4.07	95.7

Data were truncated at approximately $I/\sigma I \geq 2.0$ and $CC_{1/2} \geq 0.75$.

Table S4. Refined bond lengths (unrestrained) for the inorganic chains

Bond	Bond length (Å)	SCXRD bond length (Å)	Bond details
Al1–O6	1.885(7)	1.901[a]	Al–O–P
Al1–O8	1.930(6)	1.901[a]	Al–O–P
Al2–O2	1.893(8)	1.901[a]	Al–O–P
Al2–O3	1.916(6)	1.901[a]	Al–O–P
Al1–O1	1.864(4)	1.882[a]	Al–O–Al
Al2–O1	1.853(6)	1.882[a]	Al–O–Al
P1–O4	1.580(8)	1.560[a]	P–O–H
P2–O7	1.560(9)	1.560[a]	P–O–H
P1–O2	1.517(6)	1.520[a]	P–O–Al
P1–O6	1.509(7)	1.520[a]	P–O–Al
P2–O3	1.513(6)	1.520[a]	P–O–Al
P2–O8	1.508(7)	1.520[a]	P–O–Al
P1–O5	1.540(7)	1.500[b]	P=O
P2–O9	1.492(8)	1.500[b]	P=O

[a] Average SCXRD bond length from similar structures[13,14,15].

[b] Idealized bond length of P=O terminal without any noncovalent interactions.

Table S5. Refined bond lengths (unrestrained) for the API molecule.

Bond	Bond length (Å)	SCXRD bond length (Å)[16]	Bond details
N1–C1	1.318(14)	1.317	imidazole ring
N1–C2	1.362(16)	1.363	imidazole ring
N2–C1	1.303(10)	1.323	imidazole ring
N2–C3	1.382(15)	1.367	imidazole ring
N2–C4	1.455(12)	1.455	chain tail
N3–C6	1.492(10)	1.495	chain tail
C2–C3	1.332(13)	1.334	imidazole ring
C4–C5	1.486(13)	1.523	chain tail
C5–C6	1.498(13)	1.506	chain tail

Table S6. Hydrogen bond lengths compared to idealized hydrogen bond lengths from X-ray and neutron diffraction.

X	H	AFIX	X–$H_{electron}$ (Å)	X–$H_{X\text{-}ray}$ (Å)	X–$H_{neutron}$ (Å)
C1	H2	44	1.15(5)	0.93	1.08
C2	H3	44	1.07(3)	0.93	1.08
C3	H4	44	1.08(4)	0.93	1.08
C4	H5A	24	1.17(5)	0.97	1.09
C4	H5B	24	1.16(3)	0.97	1.09
C5	H6A	24	1.15(4)	0.97	1.09
C5	H6B	24	1.15(3)	0.97	1.09
C6	H7A	24	1.11(4)	0.97	1.09
C6	H7B	24	1.10(4)	0.97	1.09
N1	H1	44	1.01(5)	0.86	1.01
N3	H8A	138	1.04(4)	0.89	1.03
N3	H8B	138	1.04(4)	0.89	1.03
N3	H8C	138	1.04(4)	0.89	1.03
O1	H9	--	0.90(8)	0.82	0.98
O4	H10	148	0.97(5)	0.82	0.98
O7	H11	148	1.08(6)	0.82	0.98

Hydrogen atoms were restrained in refinement using *SHELXL* with the AFIX command, allowing the X-H distance to be refined. Idealized bond lengths for X-ray diffraction were obtained from *SHELXL* by constraining the X-H distance. Idealized bond lengths for neutron diffraction were obtained from *SHELXL* by constraining the X-H distance and using the command NEUT.

Refinement of C2–H3 and C3–H4 were unstable, therefore the idealized geometry was constrained using AFIX 44, whereas the X–H distance was restrained to the idealized hydrogen bond length from neutron diffraction of 1.08 Å with a sigma of 0.02 Å using the DFIX command in *SHELXL*.

References

(1) Nannenga, B. L.; Shi, D.; Leslie, A. G. W.; Gonen, T. High-Resolution Structure Determination by Continuous-Rotation Data Collection in MicroED. *Nature Methods* **2014**, *11* (9), 927–930.

(2) Cichocka, M. O.; Ångström, J.; Wang, B.; Zou, X.; Smeets, S. High-Throughput Continuous Rotation Electron Diffraction Data Acquisition via Software Automation. *J. Appl. Cryst.* **2018**, *51* (6), 1652–1661.

(3) Kabsch, W. XDS. *Acta Cryst D, Acta Cryst Sect D, Acta Crystallogr. D,* **2010**, *66* (2), 125–132.

(4) Sheldrick, G. M. SHELXT – Integrated Space-Group and Crystal-Structure Determination. *Acta Cryst A* **2015**, *71* (1), 3–8.

(5) Sheldrick, G. M. Crystal Structure Refinement with SHELXL. *Acta Cryst. C* **2015**, *71* (1), 3–8.

(6) Hübschle, C. B.; Sheldrick, G. M.; Dittrich, B. ShelXle: A Qt Graphical User Interface for SHELXL. *J. Appl. Cryst.* **2011**, *44* (6), 1281–1284.

(7) Cheng, S.; Tzeng, J.-N.; Hsu, B.-Y. Synthesis and Characterization of A Novel Layered Aluminophosphate of Kanemite-like Structure. *Chem. Mater.* **1997**, *9* (8), 1788–1796.

(8) Meinhold, R. H.; Tapp, N. J. An NMR Study of the Reaction of Water with $AlPO_4$-5. *J. Chem. Soc., Chem. Commun.* **1990**, 219–220.

(9) Eduok, U.; Ohaeri, E.; Szpunar, J. Conversion of Imidazole to N-(3-Aminopropyl)Imidazole toward Enhanced Corrosion Protection of Steel in Combination with Carboxymethyl Chitosan Grafted Poly(2-Methyl-1-Vinylimidazole). *Ind. Eng. Chem. Res.* **2019**, *58* (17), 7179–7192.

(10) Paul, G.; Bisio, C.; Braschi, I.; Cossi, M.; Gatti, G.; Gianotti, E.; Marchese, L. Combined Solid-State NMR, FT-IR and Computational Studies on Layered and Porous Materials. *Chemical Society Reviews* **2018**, *47* (15), 5684–5739.

(11) Ma, Y.; Li, N.; Xiang, S.; Guan, N. IR and Raman Investigation of One-Dimensional and Three-Dimensional Aluminophosphite. *J. Phys. Chem. C* **2007**, *111* (49), 18361–18366.

(12) Parise, J. B.; Day C. S. The structure of trialuminium tris(orthophosphate) hydrate, $AlPO_4$-21, with clathrated ethylenediamine, $Al_3(PO_4)_3{\bullet}C_2H_8N_2{\bullet}H_2O$, and pyrrolidine, $Al_3(PO_4)_3{\bullet}C_4H_9N{\bullet}H_2O$. *Acta Cryst.* **1985**, *C41*, 515-520.

(13) Chen, W.-H.; Xiang, Y.; Chen, J.-Z.; Zeng, Q.-X. Poly[Propane-1,2-Diammonium [μ_2-Hydroxido-Di-μ2-Phospho-nato-Aluminium(III)] Monohydrate]. *Acta Cryst. E* **2007**, *63* (9), m2401.

(14) Li, N.; Xiang, S. Hydrothermal Synthesis and Crystal Structure of Two Novel Aluminophosphites Containing Infinite Al–O–Al Chains. *J. Mater. Chem.* **2002**, *12* (5), 1397–1400.

(15) Harvey, H. G.; Teat, S. J.; Tang, C. C.; Cranswick, L. M.; Attfield, M. P. Synthesis and Characterization of Three Novel Cation-Containing ($NH_4^+/C_3H_7NH_3^+/NH_3^+C_2H_4NH_3^+$) Aluminum Diphosphonates. *Inorg. Chem.* **2003**, *42* (7), 2428–2439.

(16) Li, Y. Y.; Lin, C. K.; Zheng, G. L.; Cheng, Z. Y.; You, H.; Wang, W. D.; Lin, J. Novel 〈110〉-Oriented Organic−Inorganic Perovskite Compound Stabilized by N-(3-Aminopropyl)Imidazole with Improved Optical Properties. *Chem. Mater.* **2006**, *18* (15), 3463–3469.
